# Comprehensive analysis of molecular, physiological, and functional biomarkers of aging with neurological diseases using Mendelian randomization

**DOI:** 10.1007/s11357-024-01334-6

**Published:** 2024-09-13

**Authors:** Yume Imahori, Chenxi Qin, Bowen Tang, Sara Hägg

**Affiliations:** The Department of Medical Epidemiology and Biostatistics, Karolinska Institutet, Stockholm, Sweden

**Keywords:** Biological age, Neurological disease, Dementia, Mendelian randomization

## Abstract

An increasing burden of neurological diseases (NDs) has been a public health challenge in an aging society. Age, especially biological age, is the most important risk factor for NDs. Identification of biomarkers of aging to capture NDs might lead to a better understanding of the underlying mechanisms of pathological brain aging and the implementation of effective intervention. We conducted a comprehensive two-sample Mendelian Randomization (MR) study to investigate the association between various biomarkers of aging and three leading causes of NDs: Alzheimer’s disease (AD), vascular dementia (VaD), and ischemic stroke. Publicly available GWAS summary statistics on people from European ancestry were obtained for six molecular biomarkers, two physiological biomarkers, and eight functional biomarkers, and three NDs. Genetic variants serving as instrumental variables (IVs) were identified for each biomarker. The MR analysis included inverse variance weighted (IVW), weighted median, MR-Egger, and MR-PRESSO. We found that short telomere length and decrease in appendicular lean mass were associated with an increased risk for AD (OR IVW = 1.12 per 1SD decrease, 95% confidence interval 1.02–1.22, and OR IVW = 1.11, 1.06–1.16, respectively), whereas high frailty index showed a protective effect for AD. Accelerated BioAge appeared to be associated with increased risk for ischemic stroke (OR IVW = 1.3 per year in BioAge acceleration, 95% CI 1.19–1.41). Our findings implied a causal association of short telomere length and a decrease in appendicular lean mass with an increased risk for AD, while BioAge appeared to be a good biomarker for ischemic stroke. Further studies are needed to validate these associations and explore underlying mechanisms.

**Supplementary Information:**

The online version contains supplementary material available at 10.1007/s11357-024-01334-6.

## Introduction

The world has experienced an unprecedented demographic shift toward an aging society with a dramatic increase in life expectancy. Despite this increase in life expectancy, the improvements in health span, defined as lifespan without chronic diseases and disability, have lagged behind. This hinders us from fully enjoying extended lifespans [1, 2]. Therefore, expanding the health span is a public health priority.

Age is the most important risk factor for various age-related chronic diseases and disability [3, 4]. However, the aging process varies widely in individuals, and chronological age often fails to capture underlying molecular damage and functional decline accurately. Thus, the evaluation of biological age, which reflects age-related biological changes, is becoming more important [5]. Unlike chronological age, biological age could be modifiable, providing tools to identify possible anti-aging interventions and treatments. Therefore, biomarkers to assess biological age have been intensely sought out in the past decades [6].

Numerous biomarkers of aging have been proposed. According to the Biomarkers of Aging Consortium’s proposal, these biomarkers can be broadly categorized into three groups based on what they measure: molecular biomarkers based on omics data, physiological biomarkers to measure functional performance, and digital biomarkers [5]. Particularly, the molecular-based group constitutes the largest group due to the recent advancement of molecular biology. At the molecular level, 12 hallmarks of aging have been identified as underlying mechanisms of the aging process [7]. Despite the increasing number of biomarkers, there is no gold standard biomarker of aging. Many aging researchers think that no single biomarker can capture all aspects of aging due to the multi-dimensional and complicated nature of the aging process. It is more likely that different biomarkers reflect distinct aging aspects across various tissues and organs.

Among age-related chronic diseases, dementia and other neurological diseases (NDs) are some of the most common and feared ones, consisting of 19.5% of total deaths in the EU [8]. Eight hallmarks of ND have been proposed as drivers of pathological neurological aging [9]. These hallmarks largely overlap with those of aging but have some distinct characteristics [7]. For example, genetic instability and DNA/RNA damage are considered primary triggers for all other hallmarks, driving both aging and neurodegeneration. Furthermore, the impairment of mitochondria, crucial for cellular energy production, appears to accelerate both aging and neurogenerative aging [7, 10, 11]. Among ND hallmarks, pathological protein accumulation and synaptic function are crucial, characterizing various NDs. This implies that ND has its own biomarkers that most accurately capture pathological neurological aging. Recently, some observational studies showed biomarkers of aging, such as the epigenetic clock based on DNA methylation data and biological age derived from routinely measured clinical biomarkers, predict NDs, including dementia [12–14]. However, as a recent systematic review shows, observational studies, especially large-scale prospective studies, are limited and have inconsistent results [15]. Besides, observational studies are susceptible to confounding and bias. Moreover, most studies use one type of biomarker at a time, which makes the comparison of various biomarkers difficult in terms of their ability to predict NDs. Mendelian randomization (MR) is an analytical approach to investigate the association using genetic variants as instrumental variables, mitigating concerns over confounding and reverse causation [16]. The use of MR may strengthen existing evidence through triangulation of evidence.

The aim of our study is to investigate the associations of various biomarkers of aging, including molecular, physiological, and functional biomarkers, with three common NDs: Alzheimer’s disease (AD), vascular dementia (VaD), and ischemic stroke, using the MR approach to provide evidence for putative causal mechanisms of biological aging effects on NDs. Our study seeks to strengthen existing evidence and shed some light on the underlying mechanism.

## Methods

### Study design

We used publicly available GWAS summary statistics for biomarkers of aging and NDs. Ethical approval was obtained in all original studies. A summary of study data sources is provided in Table 1. The paper follows reporting according to the STROBE-MR guidelines [17].

**Table 1:** Information on the studies and consortia from which genetic association data were obtained

Phenotype	Study or consortium	Ancestry	Cases/controls	Case definition	Unit
*Molecular*					
Telomere length [19]	UK Biobank	EUR	472,174	Peripheral leukocyte TL	SD
HannumAge [20]	Meta-analysis of 28 dataset	EUR	34,710	Epigenetic clock trained on chronological age	year
IEAA [20]	Meta-analysis of 28 dataset	EUR	34,710	Epigenetic clock trained on chronological age	year
PhenoAge [20]	Meta-analysis of 28 dataset	EUR	34,710	Epigenetic clock trained on PhenoAge(clinical)	year
GrimAge [20]	Meta-analysis of 28 dataset	EUR	34,710	Epigenetic clock trained on mortality	year
mtDNA-CN [21]	CHARGE, UK Biobank	EUR	465,809	Mitochondria copy number	
*Physiological*					
BioAge (clinical) [22]	UK Biobank	EUR	379,703	Composite clinical measures derived from seven clinical markers which is associated with chronological age	Year
PhenoAge (clinical) [22]	UK Biobank	EUR	379,703	Composite clinical measures derived from nine clinical markers and chronological age which is associated with mortality	Year
*Functional*					
Handgrip	UK Biobank	EUR	461,089	Assessed by squeezing the dynamometer as hard as possible	SD
Appendicular lean mass [23]	UK Biobank	EUR	450,243	Skeletal muscle mass of limbs assessed by the bioimpedance analysis approach	SD
FVC [24]	UK Biobank	EUR	421,986	The highest measurement deemed acceptable by the investigator	SD
FEV_1_ [24]	UK Biobank	EUR	421,986	The highest measurement deemed acceptable by the investigator	SD
Retinal eye clock [25]	UK Biobank	EUR	45,444	The EyePACS dataset was trained	Year
Cognitive performance [26]	COGENT, UK Biobank	EUR	257,841	The published result of the COGENT (derived from more than three neuropsychological test) and the UK Biobank results based on verbal numerical reasoning test were meta-analyzed	SD
Brain age gap [27]	UK Biobank	EUR	28,104	T1-weighted brain MRI images	Year
Frailty index [28]	UK Biobank, Swedish TwinGene	EUR	164,610	Estimated based on 49 variables about self-reported physiological and mental health condition collected at baseline	
*Positive/negative control*					
Parental lifespan [29]	UK Biobank	EUR	1,012,240	Participants completed a questionnaire which included questions on adaptation status, parental age, parental death	Year
Skin color	UK Biobank	EUR	456,692	Self-reported data coded with a scale (1 = very fair, 2 = fair, 3 = light olive, 4 = dark olive, 5 = brown, 6 = black)	NA
*Outcome*					
Alzheimer’s disease [30]	EADB, UK Biobank	EUR	111,326 (clinically diagnosed 39,106, proxy-case 46,828)/401,577	Clinically diagnosed: Cases were mainly ascertained based on clinical criteria or diagnosis from memory clinics, although the methods varied among studies Proxy-case: participants who reported at least one biological relative affected with dementia were categorized as proxy-cases	Log(OR)
Vascular dementia [31, 32]	FinnGen	EUR	881/211,508	Identified from nation-wide health registries using ICD-10 codes	Log(OR)
Ischemic stroke [33]	MEGASTROKE	EUR	40,585/406,111	Any ischemic stroke. The method to identify the case varies among studies, including clinical symptom, CT/MRI imaging, medical record review, cases from register, or death register	Log(OR)

Abbreviations: *CHARGE*, Cohorts for Heart and Aging Research in Genomic Epidemiology; *EADB*, The European Alzheimer & Dementia Biobank ; *EUR*, European population; *FVC*, forced vital capacity; *FEV*_*1*_, forced expiratory volume in 1 second; *mtDNA-CN*, mitochondrial DNA copy number; *OR*, odds ratio; *SD*, standard deviation

**Fig. 1:**
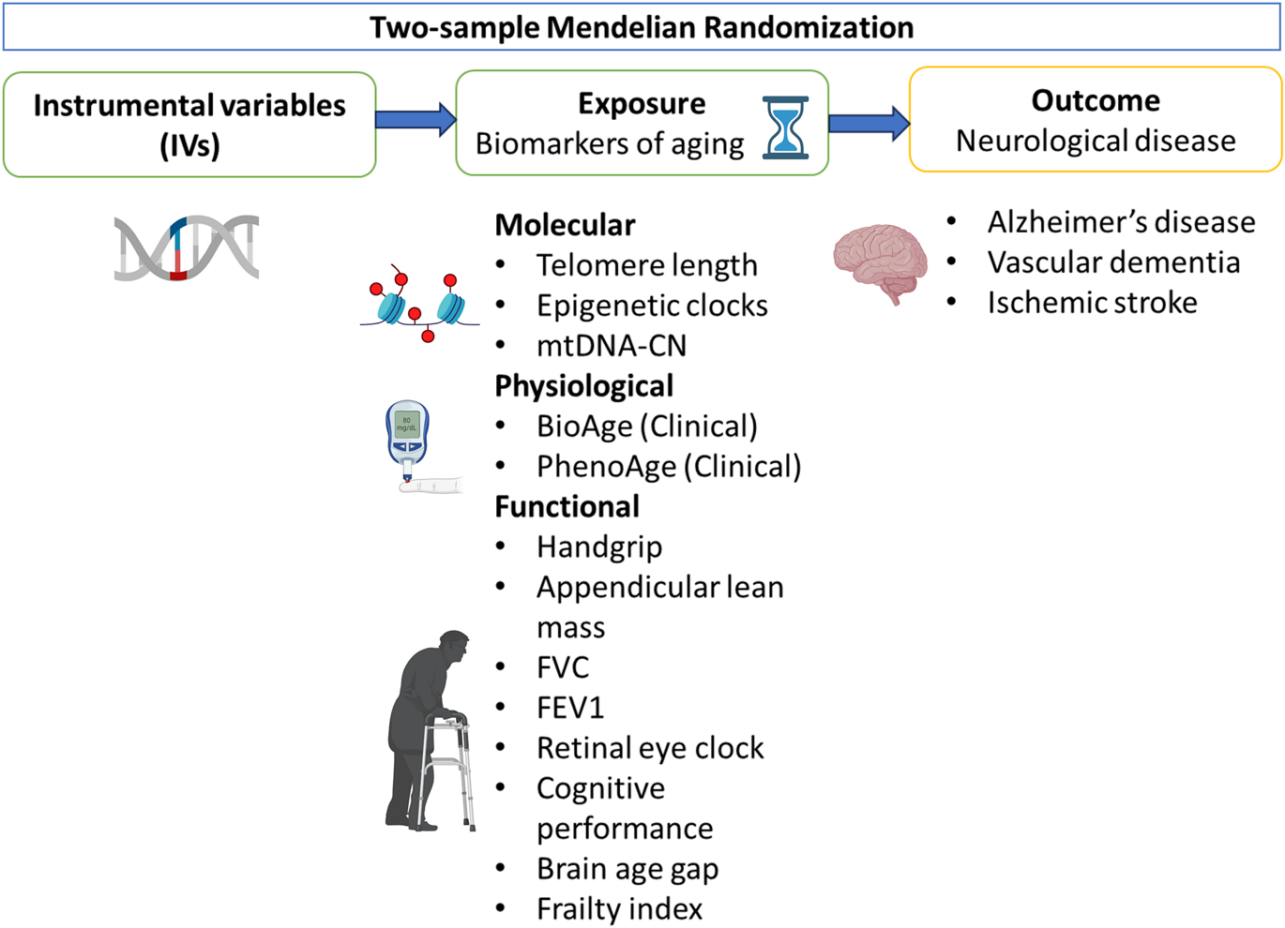
A framework of the two-sample Mendelian randomization for the effect of genetically predicted biomarkers of aging on neurological diseases. FEV1, forced expiratory volume; FVC, forced vital capacity; mtDNA-CN, mitochondrial DNA copy number

We conducted a two-sample MR to investigate the association between multiple biomarkers of aging and three NDs. As exposures, we used six molecular biomarkers, two physiological biomarkers, and eight functional biomarkers. Genetic variants that are used as instrumental variables (IVs) were identified in the GWAS dataset for each biomarker. To obtain unbiased causal estimates in MR, IVs need to satisfy three key assumptions. First, IVs need to be associated with the exposure of interest (the relevance assumption). Secondly, IVs should not share a common cause with the outcome (the independent assumption). Finally, IVs should not affect the outcome except through the exposure (the exclusion restriction assumption) [18]. A framework of our study design is presented in Fig. 1.

### Genetic instruments for biomarkers of aging

We obtained publicly available GWAS summary statistics for various biomarkers of aging. We categorized biomarkers into three groups: molecular biomarkers based on omics, physiological biomarkers based on clinical data, and functional biomarkers based on functional performance. Molecular biomarkers include telomere length [19], four types of epigenetic clocks derived from DNA methylation (HannumAge,

IEAA, PhenoAge, GrimAge) [20], and mitochondria DNA copy number [21]. As physiological biomarkers, we obtained BioAge acceleration and PhenoAge acceleration [22]. Functional biomarkers included handgrip, appendicular lean mass, two lung function measures (FVC, forced vital capacity; FEV_1_, forced expiratory volume in 1 s), cognitive performance [26], retinal eye clock [25], brain age gap [27], and frailty index [28]. The short explanation of each biomarker is presented in Table 2.

**Table 2:** A list of biomarkers of aging

Biomarker of aging	Biomarker type
*Molecular biomarkers*	
Telomere length [19]	Nucleoprotein complexes located at the ends of linear chromosomes which protect them from DNA damage, which shorten with age
HannumAge [34]	The first generation of epigenetic clock based on DNA methylation (71 CpGs in leukocytes) associated with chronological age
IEAA [35]	The first generation of epigenetic clock based on DNA methylation (353 CpGs) regressed on chronological age and cell count information
PhenoAge [36]	The second generation of epigenetic clock based on DNA methylation associated with phenotypic age (biological age based on various clinical data and chronological age associated with mortality)
GrimAge [37]	The epigenetic clock based on DNA methylation associated with various clinical data and smoking pack-year, and regressed by time-to-death
mtDNA-CN	Mitochondria copy number
*Physiological biomarkers*	
BioAge (clinical) [38]	Composite clinical measures derived from seven clinical markers (albumin, creatinine, C-reactive protein, alkaline phosphatase, glycated hemoglobin, systolic blood pressure, total cholesterol) which are associated with chronological age
PhenoAge (clinical) [36]	Composite clinical measures derived from nine clinical markers (albumin, creatinine, glucose, C-reactive protein, lymphocyte percent, mean cell volume, red cell distribution width, alkaline phosphatase, white blood cell count) and chronological age which is associated with mortality
*Functional biomarkers*	
Handgrip [39]	The indicator of overall muscle strength assessed by squeezing the dynamometer as hard as possible
Appendicular lean mass	Skeletal muscle mass of limbs
FVC [40]	The volume delivered during an expiration made as forcefully and completely as possible starting from full inspiration assessed by spirometry
FEV_1_ [40]	The expiratory volume in the first second of an FVC maneuver
Retinal eye clock [25]	The biomarker derived from fundus image from EyePACS dataset trained with chronological age using a machine learning approach
Cognitive performance [26]	Derived from three or more neuropsychological tests in the COGENT study and score on a test of verbal-numerical reasoning which was designed to measure fluid intelligence in the UK Biobank study
Brain age gap [27]	The difference between chronological age and brain age derived from MRI brain scan imaging by machine learning approach
Frailty index [28]	The measure of frailty based on the accumulation of a number of health deficits

Abbreviations: *CpGs*, cytosine phosphate guanines; *FVC*, forced vital capacity; *FEV*_*1*_, forced expiratory volume in 1 second; *mtDNA-CN*, mitochondrial DNA copy number

For each biomarker, we identified variants with genome-wide significance (*p* < 5 × 10^−8^), and retained those independent variants as IVs (linkage disequilibrium *r*^*2*^ < 0.001) based on the European reference panel from the 1000 Genomes Project Phase 3 (Supplementary Table 1). The plausibleness of the relevant assumption was assessed using F statistics [18]. The proportion of trait variance explained by genetic instruments R^2^ was estimated using the formulae:

${R}^{2}=(2{\beta }^{2}\text{ MAF (1-MAF))}/(2{\beta }^{2} MAF \left(1-MAF\right)+2N MAF \left(1-MAF\right) S{E}^{2})$ and the F statistic was calculated using the following formulae: $F=({R}^{2} (N-2)/(1-{R}^{2})$ (where MAF = effect allele frequency, *N* = sample size, *β* = beta coefficient of the SNP, SE = standard error).

### Study outcomes

We obtained publicly available GWAS summary statistics for each ND outcome. Only genetic data on people with European ancestry was included. We obtained the summary statistics for AD from the largest AD GWAS data, with 39,106 clinical cases, 46,828 proxy cases, and 401,577 controls [30]. In this GWAS data, the results from the European Alzheimer & Dementia Biobank consortium with clinical AD cases were meta-analyzed with the UK Biobank results with proxy AD cases. Clinical AD cases were mainly ascertained through clinical criteria and/or diagnosis from memory clinics, in spite of some variations among studies in the consortium [30]. Proxy AD cases in the UK Biobank were identified when participants had at least one parent or sibling affected by dementia. For VaD, summary statistics were taken from FinnGen, comprising 881 cases and 221,508 controls. Cases were identified from nationwide health registries using ICD-10 codes [32]. The summary statistics of all ischemic strokes were obtained from the MEGASTROKE consortium, including 40,585 cases and 406,111 controls in the European population (Table 1) [33]. The identification of ischemic stroke cases was mainly based on clinical symptoms and CT/MRI imaging in spite of variations among the included studies. Details of AD and stroke case identification can be found in the original articles [30, 33].

### Positive and negative control analysis

To assess the validity of selected SNPs, we conducted an MR analysis using parental lifespan as a positive control outcome, where statistically significant associations with biomarkers of aging are expected. The absence of significant association may indicate invalid IVs or low power. Furthermore, to detect potential population stratification and invalidity of IVs, we repeated the analysis using skin color as a negative control outcome, where no associations with biomarkers of aging are expected. Any statistically significant associations with this negative control would suggest potential population stratification or invalid IVs [41]. GWAS data for parental lifespan were sourced from the University of Edinburgh data share site (http://dx.doi.org/10.7488/ds/2463), and that for skin color was obtained using the University of Bristol’s IEU OpenGWAS API (ukb-b-19560).

### Statistical analysis

Then, we conducted a two-sample MR analysis to investigate the associations between biomarkers of aging and three NDs. The IV dataset was merged with the outcome GWAS summary. For an absent SNP in ND GWAS, a proxy SNP was sought using the European panel from the 1000 Genome Project as the reference panel (R^2^ > 0.8). Palindromic SNPs with minor allele frequency > 0.42 were excluded. As the main analysis, we used the inverse variance-weighted (IVW) method to estimate the association of biomarkers of aging with each outcome. The Cochran’s Q test was used to assess heterogeneity across the IVs.

### Sensitivity analysis

To obtain more robust MR estimates and explore violations of the exclusion restriction assumption, we conducted several sensitivity analyses, including the weighted median regression which allows up to 50% of invalid IVs [42], MR-Egger regression which allows the presence of pleiotropy [43], and MR-PRESSO which can detect and correct horizontal pleiotropy [44]. Similar effect estimates across various methods make the observed association more likely [45]. The funnel plots were visually inspected where asymmetry suggesting the presence of pleiotropy. Furthermore, due to sample overlap between the AD GWAS and GWAS for biomarkers of aging, we repeated the AD analysis by excluding the UK Biobank population from the AD GWAS.

All statistical analyses were performed in R version 4.2.3 using the TwoSampleMR package. For each MR analysis, post hoc power calculations were conducted using an online calculator tool (https://sb452.shinyapps.io/power/).

## Results

F-statistics of genetic instruments ranged from 28.0 to 1105.8 (Supplementary Table 2). The MR analysis using the negative control outcome skin color did not show evidence of population stratification for most of the biomarkers, except for cognitive performance and frailty index (Supplementary Fig. 1). Regarding the analysis using the positive control outcome (parental lifespan), all molecular biomarkers showed statistically insignificant associations although the direction of association indicated that advanced biological age was associated with shorter parental lifespan, as expected (Supplementary Fig. 2). Most functional biomarkers showed statistically significant associations in the expected direction. Regarding appendicular lean mass, FEV_1_, and retinal eye clock, the associations were insignificant in spite of expected directions.

### Biomarkers of aging and AD

**Fig. 2:**
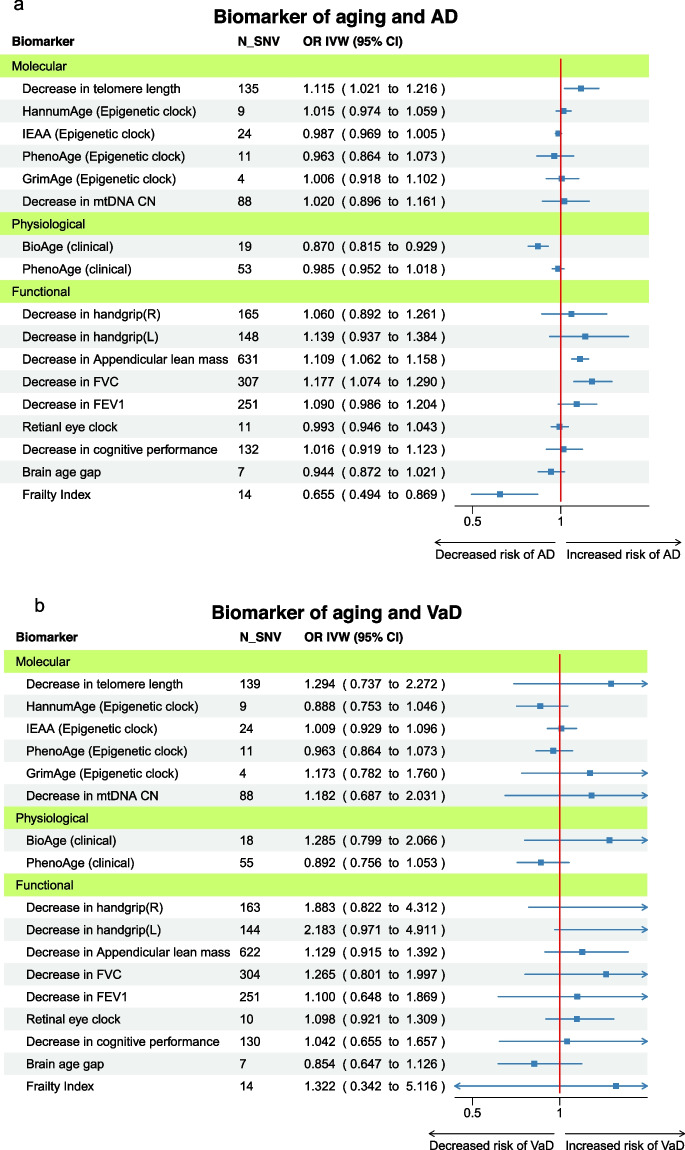

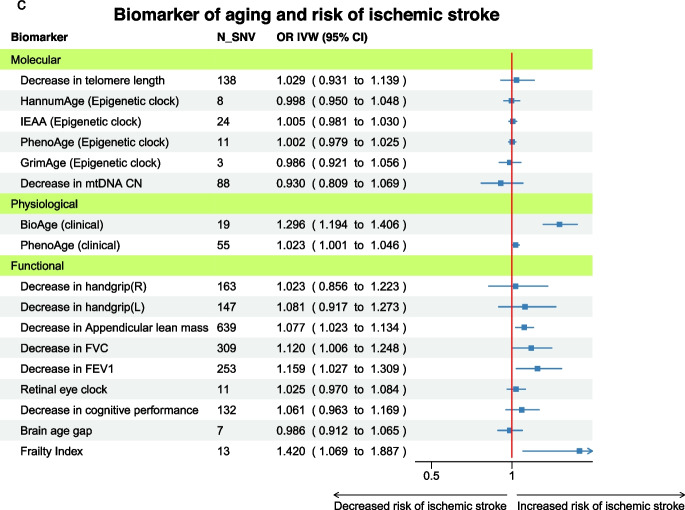
Inverse-variance weighted Mendelian randomization estimates for genetically predicted biomarkers of aging on **A** Alzheimer’s disease, **B** vascular dementia, and **C** ischemic stroke. AD, Alzheimer’s disease; CI, confidence interval; FEV1, forced expiratory volume; FVC, forced vital capacity; N_SNV, number of single-nucleotide variations; OR IVW, odds ratio inverse variance weighted; VaD, vascular dementia

Overall, we observed a positive association between advanced biological age and increased risk of AD (Fig. 2A). This association was clear in functional biomarkers but not in molecular biomarkers. Among molecular biomarkers, only short TL was associated with an increased risk of AD (OR IVW = 1.12 per 1SD increase in TL, 95% CI 1.02–1.22). Physiological biomarkers showed rather a protective effect. BioAge acceleration was associated with decreased risk of AD (OR IVW = 0.87 per year in BioAge acceleration, 95% CI 0.82–0.93). Functional biomarkers appeared to increase the risk of AD, with some evidence for appendicular lean mass (OR IVW = 1.11 per 1SD decrease in lean mass, 95% CI 1.06–1.16) and FVC (OR IVW = 1.18 per 1SD decrease in FVC, 95% CI 1.07–1.29). On the contrary, the genetic liability for a higher frailty index showed a protective effect of AD (OR IVW = 0.67, 95% CI 0.49–0.87). Post hoc power calculation suggested AD GWAS sample size is enough for most biomarkers except for some biomarkers such as GrimAge, BioAge, retinal eye clock, brain age gap, and frailty index (Supplementary Table 3).

### Biomarkers of aging and VaD

We did not find a clear trend in the association between advanced biological age and VaD using the IVW (Fig. 2B). Biomarkers showed no evidence of association with the risk of VaD, and the confidence intervals were considerably wide. Post hoc power calculation suggests that the sample size is not enough to detect the association between biomarkers of aging and VaD with 80% power (Supplementary Table 3).

### Biomarkers of aging and ischemic stroke

The results from IVW MR suggested a positive association of physiological and functional biomarkers with risk of ischemic stroke (Fig. 2C). However, no single molecular biomarkers were associated with the risk of ischemic stroke. Regarding physiological biomarkers, both BioAge and PhenoAge acceleration were associated with an increased risk of ischemic stroke (OR IVW = 1.30 per year in BioAge acceleration, 95% CI 1.19–1.41; OR IVW = 1.02 per year in PhenoAge acceleration, 95% CI 1.00–1.05). Among functional biomarkers, a decrease in lung function, both FVC and FEV_1_, and lower appendicular lean mass increased the risk of ischemic stroke (OR IVW = 1.08, 1.12, and 1.16 per 1 SE decrease, respectively).

### Sensitivity analysis

Cochran’s Q statistics suggested heterogeneity of effect estimates among SNPs for many biomarkers (Supplementary Table 2). However, the intercept of MR Egger did not indicate the presence of pleiotropy for most of the biomarkers (Supplementary Table 2). MR estimates obtained from more robust methods are presented in Supplementary Table 2. Although most of the associations became insignificant, TL, appendicular lean mass, and frailty remained significantly associated with AD in most sensitivity analyses. Regarding ischemic stroke, only the association with BioAge acceleration remained robust in sensitivity analyses.

The GWAS for biomarkers of aging we used were mostly derived from the UK Biobank participants. Because our AD GWAS includes the UKB participants, to address population overlap, we repeated the MR analysis by excluding the UKB participants from the AD GWAS (Supplementary Fig. 3). As for our main findings, appendicular lean mass stayed significantly associated with AD. Although the association of TL and frailty index became insignificant, the direction of effect remained unchanged.

## Discussion

We investigated the association of multiple biomarkers of aging with three NDs using the two-sample MR framework, and had five main findings. Firstly, a positive association of advanced biological age with increased risk of AD and ischemic stroke was observed, particularly for functional biomarkers. Notably, no molecular marker was associated with ischemic stroke. Secondly, among molecular biomarkers, short TL appeared to be associated with an increased risk of AD but not ischemic stroke. Thirdly, physiological biomarker BioAge acceleration was associated with an increased risk of ischemic stroke. Fourthly, among functional biomarkers, reduced appendicular lean mass may increase the risk of AD. Finally, an increased frailty index showed a protective effect for AD.

Our MR findings suggested that short TL may be causally related to AD. This result aligns with previous observational studies and an MR study based on 16 SNPs from a small AD GWAS [46, 47]. Regarding other molecular biomarkers, no significant associations with NDs were observed. This was unexpected, given the accumulating evidence from observational studies suggesting the association between molecular biomarkers and NDs. The discrepancy could be partially attributed to insufficient statistical power. The number of instrumental variables tended to be lower for molecular biomarkers than for functional ones, except for TL. Moreover, the sample size for GWAS data on four epigenetic clocks was smaller in comparison to GWAS data for functional biomarkers. It is possible that we could not capture the true association between molecular biomarkers and neurological diseases due to the quality of instrumental variables. For an unbiased and precise MR estimate, further studies are necessary, utilizing more valid SNPs and enhanced GWAS data.

Regarding our MR finding of the association between appendicular lean mass and AD, prospective observational studies are limited and have mixed results. In a US study with 1175 community-dwelling older people, a decrease in skeletal muscle mass estimated using bioimpedance analysis was not associated with AD/mild cognitive impairment, while grip strength was associated with AD after a 5.6-year follow-up [48]. Conversely, a small US study with 344 old people showed that total lean mass, especially appendicular lean mass, was associated with a low risk for dementia, while handgrip was not after 15 years of follow-up [49]. Taken together, skeletal muscle appears to play an important role in the development of dementia. Further prospective or MR studies of high quality are needed to confirm this finding. A high frailty index, another functional biomarker, showed a protective effect for AD, which is counter intuitive. Previous observational studies in this area are relatively limited with mixed results. Some meta-analyses of cohort studies suggest that frailty is associated with dementia and cognitive decline, which contrasts with our findings [50–52]. However, most cohort studies measure frailty using the frailty phenotype, and the frailty index is dichotomized. Additionally, studies using a continuous frailty index are not included in these meta-analyses, which might explain differences from our findings. Regarding a protective effect for AD observed in our study, selective survival might to some extent explain this. Unlike dementia, the association between a high frailty index and increased mortality has been consistently shown in previous cohort studies [53]. High mortality rates among people with a high frailty index might preclude the occurrence of dementia, leading to an apparent protective effect. However, the frailty index showed a significant association with negative control outcome, raising some concerns over the presence of population stratification and the validity of the IVs. An MR study using more valid IVs, along with a prospective cohort study with a strong design, is needed to confirm our findings.

Regarding ischemic stroke, BioAge showed a robust association with an increased risk. This finding is consistent with two recent cohort studies. In the SATSA study conducted in Sweden, participants with high BioAge acceleration at baseline were associated with a higher risk of ischemic stroke over a 20-year follow-up [12]. Similarly, the analysis of 325,870 UK Biobank participants showed that BioAge acceleration at baseline was associated with ischemic stroke, along with all-cause dementia, and vascular dementia [13]. Taken together, these findings suggest that BioAge could serve as a valuable biomarker for predicting vascular brain aging. Conversely, BioAge appeared to have a protective effect for AD in our analysis. Previous studies show that high BioAge derived using Klemera and Doubal method was associated with high mortality [38, 54, 55]. Furthermore, BioAge is derived from several cardiovascular risk factors, such as systolic blood pressure and total cholesterol, and it reflects cardiovascular health. Similar to the discussion about frailty above, this apparent protective effect might be attributed to high mortality and cardiovascular risk in people with high BioAge.

The exact biological mechanism linking telomere length with AD remains unclear. Human telomeres, located at the end of chromosomes, protect them from DNA damage and regulate gene expression changes. Progressive telomere shortening with age leads not only to DNA damage and cell senescence but also to changes in gene expression and epigenetic alterations [56]. Because DNA instability induced by telomere attrition is upstream of all aging hallmarks, it can drive all remaining hallmarks [57]. This might explain the potential association between telomere shortening and brain aging. Regarding the link between appendicular lean mass and AD, a lot of attention has been paid to the role of myokines recently. Evidence suggests that loss of proteostasis, deregulated nutrient sensing, and mitochondria dysfunction are important drivers of skeletal muscle aging and serve as aging hallmarks [58]. Skeletal muscle, which comprises 40% of the human body, is the main source of myokines, including inflammatory cytokines, brain-derived neurotrophic factor, and fibroblast growth factor 21. Myokines play various roles in maintaining bodily homeostasis, including the control of inflammation and insulin resistance. Recent evidence suggests myokines might play a vital role in the cross-talk between skeletal muscle and the brain, helping to maintain cognitive function [59]. Skeletal muscle aging leads to decreased muscle mass and strength, resulting in a lower level of myokines. This reduction in myokines may contribute to cognitive dysfunction through changes in autocrine, paracrine, and endocrine function [60]. The link between BioAge acceleration and ischemic stroke is understandable because BioAge includes some cardiovascular risk factors such as systolic blood pressure and total cholesterol [22].

We conducted a comprehensive MR analysis to investigate the association of various biomarkers of aging with three neurological diseases, identifying several potential biomarkers. Some of our findings lend support to previous observational studies through triangulation of evidence. Nevertheless, our study has several limitations. First, there was a large population overlap between our exposure GWAS and the AD GWAS, attributed to both studies including participants from the UK Biobank participants. Overlapping samples can generate biased effect estimates. However, a recent simulation study suggests that bias introduced by population overlap might not be as significant as previously anticipated [61]. Besides, eliminating overlapping samples could lead to weak statistical power [62]. Consequently, to preserve statistical power, we investigated the association between biological age and AD using overlapping samples in the main analysis and conducted a sensitivity analysis excluding overlapping samples, which did not materially alter our main findings. Second, we cannot rule out the potential presence of population stratification, which introduces confounding. Indeed, a recent research study showed geographical clustering of genetic traits in the UK, most notably educational attainment [63]. Nevertheless, our analyses using negative control outcomes did not show a strong suggestion of the presence of population stratification for the majority of the biomarkers, except for cognitive performance and frailty index. Third, some biomarkers of aging had a small number of IVs, and post hoc power calculation showed a lack of outcome GWAS sample size, especially for VaD GWAS. Due to inadequate statistical power, our findings might not accurately reflect a true association. Further MR studies are required to confirm our findings. Finally, our analysis included only participants of European ancestry due to sample size availability. GWAS studies with more diverse populations are needed to enhance our understanding of the risk mechanism of NDs.

From a clinical perspective, our findings suggest that people with short TL and decreased appendicular lean mass are at an increased risk of AD and that BioAge acceleration increases the risk of ischemic stroke. If further studies confirm causal relationships, they might help identify high-risk populations and lead to potential intervention targets. Particularly, the appendicular lean mass would be an attractive target because bioimpedance analysis is noninvasive, widely available, and inexpensive, and an intervention to prevent lean mass loss is possible.

In conclusion, our findings suggested underlying causal associations of short telomere length and decreased appendicular lean mass with an increased risk for AD, while BioAge appeared to be a good biomarker for ischemic stroke. Further studies are needed to validate these associations and explore underlying mechanisms.

## Supplementary Information

Below is the link to the electronic supplementary material.

Supplementary file1 (PDF 97 KB)

Supplementary file2 (XLSX 169 KB)

Supplementary file3 (DOCX 115 KB)
