# Supplementary material for: Comprehensive analysis of molecular, physiological, and functional biomarkers of aging with neurological diseases using Mendelian randomization
Source: GeroScience. 2024 Sep 13;47(3):2959–72. doi: 10.1007/s11357-024-01334-6 (PMC12181546; doi:10.1007/s11357-024-01334-6)

Supplementary Fig.1

Biomarker of aging and skin colour (Negative control)

Biomarker	IV	beta (95% CI)
**Molecular**		
Decrease in telomere length	143	−0.009 (−0.025 to 0.007)
HannumAge (Epigenetic clock)	9	0.001 (−0.006 to 0.008)
IEAA (Epigenetic clock)	24	−0.001 (−0.004 to 0.001)
PhenoAge (Epigenetic clock)	11	−0.002 (−0.005 to 0.001)
GrimAge (Epigenetic clock)	4	0.002 (−0.005 to 0.008)
Decrease in mtDNA CN	89	−0.004 (−0.023 to 0.015)
**Physiological**		
BioAge (clinical)	19	0.008 (−0.008 to 0.025)
PhenoAge (clinical)	57	0.004 (0.001 to 0.008)
**Functional**		
Decrease in handgrip(R)	167	−0.022 (−0.067 to 0.023)
Decrease in handgrip(L)	150	−0.002 (−0.048 to 0.044)
Decrease in Appendicular lean mass	627	−0.002 (−0.015 to 0.010)
Decrease in FVC	310	0.012 (−0.009 to 0.034)
Decrease in FEV1	255	0.006 (−0.019 to 0.032)
Retinal eye clock	11	0.018 (−0.033 to 0.069)
Decrease in cognitive performance	132	0.043 (0.023 to 0.062)
Brain age gap	7	0.005 (−0.005 to 0.016)
Frailty Index	16	0.058 (0.009 to 0.107)

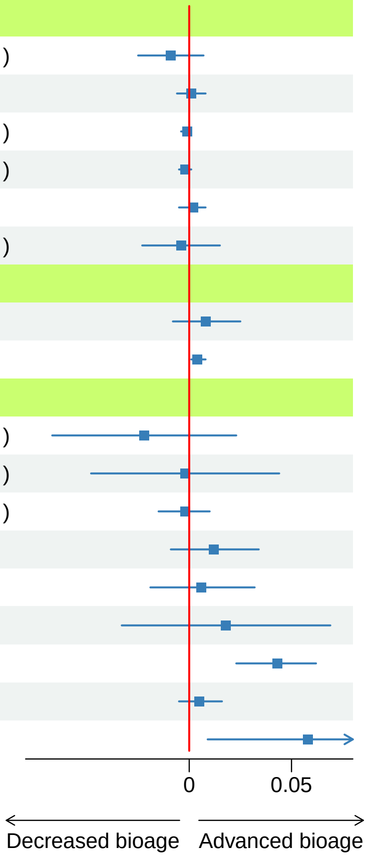

Supplementary Fig.2

Biomarker of aging and parental lifespan (positive control)

Biomarker	IV	beta (95% CI)
**Molecular**		
Decrease in telomere length	129	−0.042 (−0.114 to 0.030)
HannumAge (Epigenetic clock)	9	−0.005 (−0.030 to 0.021)
IEAA (Epigenetic clock)	24	−0.005 (−0.013 to 0.003)
PhenoAge (Epigenetic clock)	11	−0.007 (−0.015 to 0.001)
GrimAge (Epigenetic clock)	4	−0.010 (−0.040 to 0.019)
Decrease in mtDNA CN	85	0.012 (−0.045 to 0.068)
**Physiological**		
BioAge (clinical)	17	−0.145 (−0.204 to −0.086)
PhenoAge (clinical)	55	0.016 (−0.005 to 0.037)
**Functional**		
Decrease in handgrip(R)	165	−0.128 (−0.217 to −0.038)
Decrease in handgrip(L)	147	−0.145 (−0.235 to −0.055)
Decrease in Appendicular lean mass	565	−0.020 (−0.044 to 0.004)
Decrease in FVC	305	−0.070 (−0.120 to −0.020)
Decrease in FEV1	250	−0.052 (−0.109 to 0.004)
Retinal eye clock	10	0.003 (−0.016 to 0.022)
Decrease in cognitive performance	129	−0.168 (−0.222 to −0.114)
Brain age gap	7	0.012 (−0.012 to 0.037)
Frailty Index	16	−0.257 (−0.382 to −0.131)

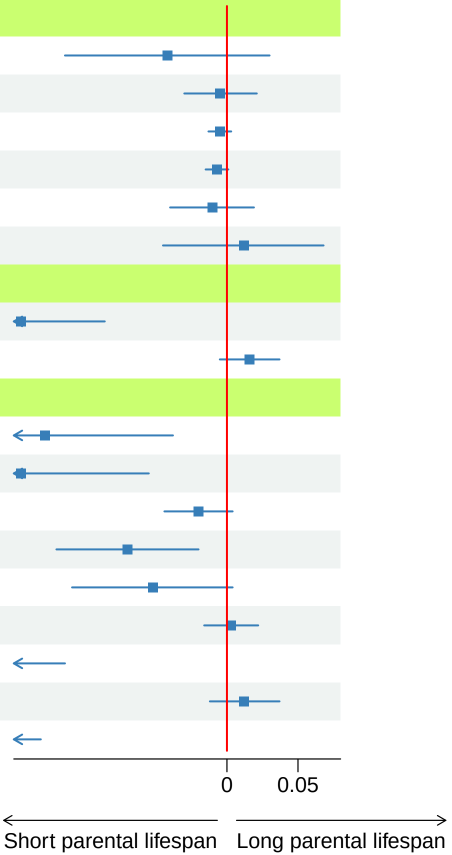

Supplementary Fig.3

Biomarker of ageing and AD excluding UKB

Biomarker	N_SNV	OR IVW (95% CI)
**Molecular**		
Decrease in telomere length	143	1.007 (0.928 to 1.094)
HannumAge (Epigenetic clock)	9	1.001 (0.995 to 1.008)
IEAA (Epigenetic clock)	24	0.997 (0.993 to 1.002)
PhenoAge (Epigenetic clock)	11	0.998 (0.993 to 1.002)
GrimAge (Epigenetic clock)	4	1.006 (0.994 to 1.018)
Decrease in mtDNA CN	88	1.044 (0.980 to 1.113)
**Physiological**		
BioAge (clinical)	20	0.999 (0.967 to 1.032)
PhenoAge (clinical)	58	0.967 (0.942 to 0.992)
**Functional**		
Decrease in handgrip(R)	165	1.033 (0.993 to 1.076)
Decrease in handgrip(L)	150	1.054 (1.009 to 1.101)
Decrease in Appendicular lean mass	649	1.024 (1.013 to 1.035)
Decrease in FVC	309	1.052 (1.016 to 1.090)
Decrease in FEV1	253	1.026 (0.977 to 1.078)
Retinal eye clock	11	0.999 (0.991 to 1.007)
Decrease in cognitive performance	132	1.020 (0.995 to 1.046)
Brain age gap	7	0.999 (0.984 to 1.014)
Frailty Index	14	0.990 (0.914 to 1.072)

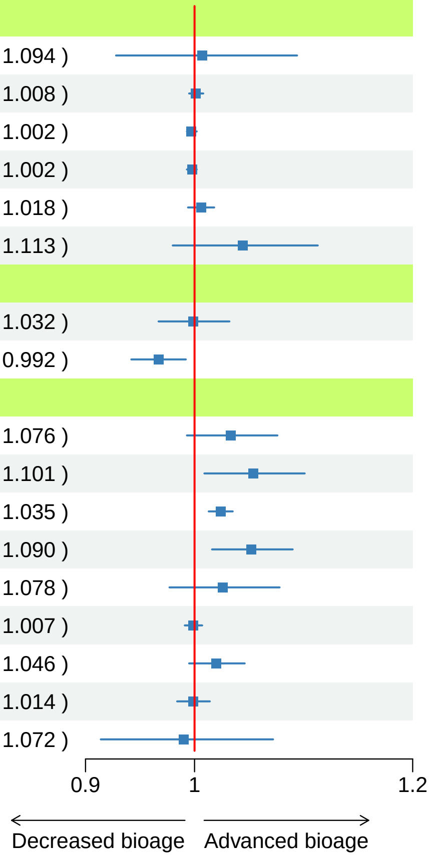

Supplement: Supplementary file 1 — Supplementary file1 (PDF 97 KB) [file 11357_2024_1334_MOESM1_ESM.pdf]
