# Supplementary material for: Comprehensive analysis of molecular, physiological, and functional biomarkers of aging with neurological diseases using Mendelian randomization
Source: GeroScience. 2024 Sep 13;47(3):2959–72. doi: 10.1007/s11357-024-01334-6 (PMC12181546; doi:10.1007/s11357-024-01334-6)
Supplement: Supplementary file 3 — Supplementary file3 (DOCX 115 KB) [file 11357_2024_1334_MOESM3_ESM.docx]

**Supplementary Table 2**

**The association between biomarkers of aging and Alzheimer’s disease**

| Exposure | method | SNP | medianF | F range | OR | 95%CI | P val | Beta | SE | Q | P_Q_ | Egger intercept | P_intercept_ |
| --- | --- | --- | --- | --- | --- | --- | --- | --- | --- | --- | --- | --- | --- |
| Decrease in Telomere length | Inverse variance weighted | 135 | 61.2 | 29.8-1105.8 | 1.115 | 1.021-1.216 | **0.015** | 0.109 | 0.045 | 249.8 | **0.000** |  |  |
|  | Weighted median | 135 | 61.2 | 29.8-1105.8 | 1.173 | 1.049-1.312 | **0.005** | 0.160 | 0.057 | NA | NA |  |  |
|  | MR Egger | 135 | 61.2 | 29.8-1105.8 | 1.157 | 0.985-1.359 | 0.079 | 0.146 | 0.082 | 249.3 | **0.000** | -0.001 | 0.590 |
|  | Weighted mode | 135 | 61.2 | 29.8-1105.8 | 1.160 | 1.015-1.326 | **0.031** | 0.149 | 0.068 | NA | NA |  |  |
|  | MR PRESSO | 133 |  |  | 1.087 | 1.007-1.173 | **0.036** | 0.083 | 0.039 |  |  |  |  |
| Hanum age acceleration | Inverse variance weighted | 9 | 37.6 | 30.8-98.9 | 1.015 | 0.974-1.059 | 0.469 | 0.015 | 0.021 | 21.2 | **0.007** |  |  |
|  | Weighted median | 9 | 37.6 | 30.8-98.9 | 1.013 | 0.974-1.053 | 0.529 | 0.012 | 0.020 | NA | NA |  |  |
|  | MR Egger | 9 | 37.6 | 30.8-98.9 | 1.061 | 0.902-1.247 | 0.498 | 0.059 | 0.082 | 20.3 | **0.005** | -0.012 | 0.600 |
|  | Weighted mode | 9 | 37.6 | 30.8-98.9 | 0.995 | 0.916-1.081 | 0.915 | -0.005 | 0.042 | NA | NA |  |  |
|  | MR PRESSO | 8 |  |  | 0.992 | 0.958-1.027 | 0.678 | -0.008 | 0.018 |  |  |  |  |
| IEAA age acceleration | Inverse variance weighted | 24 | 47.4 | 31.1-239.7 | 0.987 | 0.969-1.005 | 0.158 | -0.013 | 0.009 | 37.1 | **0.032** |  |  |
|  | Weighted median | 24 | 47.4 | 31.1-239.7 | 1.019 | 0.972-1.068 | 0.448 | 0.019 | 0.024 | 34.0 | **0.050** |  |  |
|  | MR Egger | 24 | 47.4 | 31.1-239.7 | 1.000 | 0.978-1.021 | 0.973 | -0.000 | 0.011 | NA | NA | -0.009 | 0.168 |
|  | Weighted mode | 24 | 47.4 | 31.1-239.7 | 1.003 | 0.973-1.034 | 0.846 | 0.003 | 0.016 | NA | NA |  |  |
|  | MR PRESSO | NA |  |  |  |  |  |  |  |  |  |  |  |
| PhenoAge acceleration | Inverse variance weighted | 11 | 44.9 | 31.8-89.4 | 0.990 | 0.973-1.008 | 0.277 | -0.010 | 0.009 | 4.2 | 0.937 |  |  |
|  | Weighted median | 11 | 44.9 | 31.8-89.4 | 0.981 | 0.960-1.003 | 0.092 | -0.019 | 0.011 | NA | NA |  |  |
|  | MR Egger | 11 | 44.9 | 31.8-89.4 | 0.993 | 0.944-1.045 | 0.789 | -0.007 | 0.026 | 4.2 | 0.898 | -0.001 | 0.915 |
|  | Weighted mode | 11 | 44.9 | 31.8-89.4 | 0.977 | 0.944-1.012 | 0.232 | -0.023 | 0.018 | NA | NA |  |  |
|  | MR PRESSO | NA |  |  |  |  |  |  |  |  |  |  |  |
| GrimAge acceleration | Inverse variance weighted | 4 | 35.9 | 31.1-45.5 | 1.006 | 0.918-1.102 | 0.902 | 0.006 | 0.047 | 13.0 | **0.005** |  |  |
|  | Weighted median | 4 | 35.9 | 31.1-45.5 | 1.031 | 0.972-1.093 | 0.314 | 0.030 | 0.030 | NA | NA |  |  |
|  | MR Egger | 4 | 35.9 | 31.1-45.5 | 3.418 | 1.584-7.377 | 0.089 | 1.229 | 0.392 | 2.2 | 0.331 | -0.228 | 0.089 |
|  | Weighted mode | 4 | 35.9 | 31.1-45.5 | 1.049 | 0.978-1.125 | 0.277 | 0.047 | 0.036 | NA | NA |  |  |
|  | MR PRESSO | 2 |  |  | 1.024 | 0.975-1.076 | 0.518 | 0.024 | 0.025 |  |  |  |  |
| Decrease in mtDNA copy number | Inverse variance weighted | 88 | 45.9 | 29.8-634.2 | 1.020 | 0.896-1.161 | 0.767 | 0.020 | 0.066 | 197.6 | **0.000** |  |  |
|  | Weighted median | 88 | 45.9 | 29.8-634.2 | 0.999 | 0.860-1.160 | 0.985 | -0.001 | 0.076 | NA | NA |  |  |
|  | MR Egger | 88 | 45.9 | 29.8-634.2 | 0.967 | 0.748-1.249 | 0.795 | -0.034 | 0.131 | 197.1 | **0.000** | 0.002 | 0.635 |
|  | Weighted mode | 88 | 45.9 | 29.8-634.2 | 0.949 | 0.748-1.205 | 0.670 | -0.052 | 0.122 | NA | NA |  |  |
|  | MR PRESSO | 84 |  |  | 1.019 | 0.911-1.141 | 0.739 | 0.019 | 0.057 |  |  |  |  |
| BioAgeAcceleration (clinical) | Inverse variance weighted | 19 | 42.7 | 33.0-114.2 | 0.870 | 0.815-0.929 | **0.000** | -0.139 | 0.033 | 19.6 | 0.353 |  |  |
|  | Weighted median | 19 | 42.7 | 33.0-114.2 | 0.878 | 0.800-0.964 | **0.007** | -0.130 | 0.048 | NA | NA |  |  |
|  | MR Egger | 19 | 42.7 | 33.0-114.2 | 0.908 | 0.755-1.091 | 0.315 | -0.097 | 0.094 | 19.4 | 0.308 | -0.003 | 0.634 |
|  | Weighted mode | 19 | 42.7 | 33.0-114.2 | 0.891 | 0.784-1.013 | 0.095 | -0.115 | 0.065 | NA | NA |  |  |
|  | MR PRESSO | NA |  |  |  |  |  |  |  |  |  |  |  |
| PhenoAgeclAcceleration (clinical) | Inverse variance weighted | 53 | 46.5 | 30.4-144.6 | 0.985 | 0.952-1.018 | 0.367 | -0.015 | 0.017 | 271.8 | **0.000** |  |  |
|  | Weighted median | 53 | 46.5 | 30.4-144.6 | 1.000 | 0.977-1.023 | 0.973 | -0.000 | 0.012 | NA | NA |  |  |
|  | MR Egger | 53 | 46.5 | 30.4-144.6 | 0.956 | 0.837-1.091 | 0.505 | -0.045 | 0.068 | 270.7 | **0.000** | 0.005 | 0.649 |
|  | Weighted mode | 53 | 46.5 | 30.4-144.6 | 1.002 | 0.954-1.053 | 0.928 | 0.002 | 0.025 | NA | NA |  |  |
|  | MR PRESSO | 52 |  |  | 0.993 | 0.873-1.014 | 0.517 | -0.007 | 0.011 |  |  |  |  |
| Decrease in Handgrip (right) | Inverse variance weighted | 165 | 37.9 | 29.7-231.7 | 1.060 | 0.892-1.261 | 0.507 | 0.059 | 0.088 | 325.5 | **0.000** |  |  |
|  | Weighted median | 165 | 37.9 | 29.7-231.7 | 1.172 | 0.965-1.423 | 0.109 | 0.159 | 0.099 | NA | NA |  |  |
|  | MR Egger | 165 | 37.9 | 29.7-231.7 | 1.486 | 0.792-2.790 | 0.219 | 0.396 | 0.321 | 323.1 | **0.000** | -0.004 | 0.276 |
|  | Weighted mode | 165 | 37.9 | 29.7-231.7 | 1.107 | 0.718-1.708 | 0.646 | 0.102 | 0.221 | NA | NA |  |  |
|  | MR PRESSO | 162 |  |  | 1.141 | 0.980-1.327 | 0.091 | 0.131 | 0.077 |  |  |  |  |
| Decrease in Handgrip (left) | Inverse variance weighted | 148 | 39.0 | 29.7-191.5 | 1.139 | 0.937-1.384 | 0.191 | 0.130 | 0.099 | 332.4 | **0.000** |  |  |
|  | Weighted median | 148 | 39.0 | 29.7-191.5 | 1.191 | 0.971-1.460 | 0.093 | 0.175 | 0.104 | NA | NA |  |  |
|  | MR Egger | 148 | 39.0 | 29.7-191.5 | 1.220 | 0.587-2.536 | 0.595 | 0.199 | 0.373 | 332.3 | 0.000 | -0.001 | 0.848 |
|  | Weighted mode | 148 | 39.0 | 29.7-191.5 | 1.224 | 0.748-2.003 | 0.422 | 0.202 | 0.251 | NA | NA |  |  |
|  | MR PRESSO | 144 |  |  | 1.146 | 0.972-1.352 | 0.106 | 0.137 | 0.084 |  |  |  |  |
| Decrease in Appendicular lean mass | Inverse variance weighted | 631 | 64.0 | 29.1-1072.6 | 1.109 | 1.062-1.158 | **0.000** | 0.104 | 0.022 | 1029.1 | **0.000** |  |  |
|  | Weighted median | 631 | 64.0 | 29.1-1072.6 | 1.108 | 1.045-1.174 | **0.001** | 0.102 | 0.030 | NA | NA |  |  |
|  | MR Egger | 631 | 64.0 | 29.1-1072.6 | 1.175 | 1.062-1.300 | **0.002** | 0.161 | 0.052 | 1026.6 | **0.000** | -0.001 | 0.219 |
|  | Weighted mode | 631 | 64.0 | 29.1-1072.6 | 1.057 | 0.925-1.208 | 0.414 | 0.056 | 0.068 | NA | NA |  |  |
|  | MR PRESSO | 626 |  |  | 1.104 | 1.061-1.149 | **0.000** | 0.099 | 0.020 |  |  |  |  |
| Decrease in Lung function (FVC) | Inverse variance weighted | 307 | 45.8 | 29.9-456.6 | 1.177 | 1.074-1.290 | **0.000** | 0.163 | 0.047 | 518.7 | **0.000** |  |  |
|  | Weighted median | 307 | 45.8 | 29.9-456.6 | 1.193 | 1.065-1.337 | **0.002** | 0.177 | 0.058 | NA | NA |  |  |
|  | MR Egger | 307 | 45.8 | 29.9-456.6 | 1.115 | 0.870-1.430 | 0.390 | 0.109 | 0.127 | 518.3 | **0.000** | 0.001 | 0.649 |
|  | Weighted mode | 307 | 45.8 | 29.9-456.6 | 0.959 | 0.665-1.383 | 0.822 | -0.042 | 0.187 | NA | NA |  |  |
|  | MR PRESSO | 305 |  |  | 1.227 | 1.126-1.337 | **0.000** | 0.204 | 0.044 |  |  |  |  |
| Decrease in Lung function (FEV1) | Inverse variance weighted | 251 | 44.2 | 29.7-270.6 | 1.090 | 0.986-1.204 | 0.093 | 0.086 | 0.051 | 413.3 | **0.000** |  |  |
|  | Weighted median | 251 | 44.2 | 29.7-270.6 | 1.039 | 0.919-1.174 | 0.539 | 0.038 | 0.062 | NA | NA |  |  |
|  | MR Egger | 251 | 44.2 | 29.7-270.6 | 1.191 | 0.879-1.613 | 0.260 | 0.175 | 0.155 | 412.7 | **0.000** | -0.001 | 0.543 |
|  | Weighted mode | 251 | 44.2 | 29.7-270.6 | 0.909 | 0.642-1.286 | 0.589 | -0.096 | 0.177 | NA | NA |  |  |
|  | MR PRESSO | 249 |  |  | 1.119 | 1.019-1.228 | **0.020** | 0.112 | 0.048 |  |  |  |  |
| Retinal eye clock | Inverse variance weighted | 11 | 32.6 | 28.0-217.7 | 0.993 | 0.946-1.043 | 0.785 | -0.007 | 0.025 | 32.0 | **0.000** |  |  |
|  | Weighted median | 11 | 32.6 | 28.0-217.7 | 1.000 | 0.964-1.037 | 0.995 | -0.000 | 0.019 | NA | NA |  |  |
|  | MR Egger | 11 | 32.6 | 28.0-217.7 | 0.986 | 0.859-1.133 | 0.852 | -0.014 | 0.071 | 32.0 | **0.000** | 0.002 | 0.920 |
|  | Weighted mode | 11 | 32.6 | 28.0-217.7 | 1.003 | 0.965-1.043 | 0.890 | 0.003 | 0.020 | NA | NA |  |  |
|  | MR PRESSO | 10 |  |  | 0.993 | 0.957-1.030 | 0.706 | -0.007 | 0.019 |  |  |  |  |
| Decrease in Cognitive performance | Inverse variance weighted | 132 | 38.0 | 29.8-125.3 | 1.016 | 0.919-1.123 | 0.759 | 0.016 | 0.051 | 239.7 | **0.000** |  |  |
|  | Weighted median | 132 | 38.0 | 29.8-125.3 | 1.049 | 0.933-1.180 | 0.423 | 0.048 | 0.060 | NA | NA |  |  |
|  | MR Egger | 132 | 38.0 | 29.8-125.3 | 0.911 | 0.584-1.420 | 0.681 | -0.093 | 0.227 | 239.3 | **0.000** | 0.002 | 0.622 |
|  | Weighted mode | 132 | 38.0 | 29.8-125.3 | 1.007 | 0.768-1.320 | 0.961 | 0.007 | 0.138 | NA | NA |  |  |
|  | MR PRESSO | 128 |  |  | 1.022 | 0.938-1.114 | 0.617 | 0.022 | 0.044 |  |  |  |  |
| Brain Age Gap | Inverse variance weighted | 7 | 33.1 | 30.3-94.3 | 0.944 | 0.872-1.021 | 0.146 | -0.058 | 0.040 | 28.0 | **0.000** |  |  |
|  | Weighted median | 7 | 33.1 | 30.3-94.3 | 0.964 | 0.911-1.020 | 0.201 | -0.037 | 0.029 | NA | NA |  |  |
|  | MR Egger | 7 | 33.1 | 30.3-94.3 | 0.723 | 0.555-0.942 | 0.062 | -0.324 | 0.135 | 15.4 | **0.009** | 0.055 | 0.098 |
|  | Weighted mode | 7 | 33.1 | 30.3-94.3 | 0.971 | 0.893-1.057 | 0.524 | -0.029 | 0.043 | NA | NA |  |  |
|  | MR PRESSO | 5 |  |  | 0.975 | 0.939-1.013 | 0.269 | -0.025 | 0.019 |  |  |  |  |
| Frailty | Inverse variance weighted | 14 | 32.9 | 30.0-119.1 | 0.655 | 0.494-0.869 | **0.003** | -0.423 | 0.144 | 24.6 | **0.026** |  |  |
|  | Weighted median | 14 | 32.9 | 30.0-119.1 | 0.553 | 0.412-0.742 | **0.000** | -0.592 | 0.150 | NA | NA |  |  |
|  | MR Egger | 14 | 32.9 | 30.0-119.1 | 0.590 | 0.162-2.154 | 0.440 | -0.527 | 0.660 | 24.6 | **0.017** | 0.002 | 0.874 |
|  | Weighted mode | 14 | 32.9 | 30.0-119.1 | 0.545 | 0.374-0.793 | **0.007** | -0.607 | 0.191 | NA | NA |  |  |
|  | MR PRESSO | NA |  |  |  |  |  |  |  |  |  |  |  |

**The association between biomarkers of aging and vascular dementia**

| Exposure | method | SNP | medianF | F range | OR | 95%CI | Pval. | Beta | SE | Q | P_Q_ | Egger intercept | P_intercept_ |
| --- | --- | --- | --- | --- | --- | --- | --- | --- | --- | --- | --- | --- | --- |
| Decrease in Telomere length | Inverse variance weighted | 139 | 58.7 | 29.8-1105.8 | 1.294 | 0.737-2.272 | 0.370 | 0.258 | 0.287 | 320.0 | **0.000** |  |  |
|  | Weighted median | 139 | 58.7 | 29.8-1105.8 | 1.707 | 0.961-3.032 | 0.068 | 0.534 | 0.293 | NA | NA |  |  |
|  | MR Egger | 139 | 58.7 | 29.8-1105.8 | 1.391 | 0.497-3.891 | 0.530 | 0.330 | 0.525 | 319.9 | **0.000** | -0.002 | 0.869 |
|  | Weighted mode | 139 | 58.7 | 29.8-1105.8 | 1.612 | 0.828-3.136 | 0.162 | 0.477 | 0.340 | NA | NA |  |  |
|  | MR PRESSO | 138 |  |  | 1.336 | 0.900-1.983 | 0.153 | 0.290 | 0.202 |  |  |  |  |
| Hanum age acceleration | Inverse variance weighted | 9 | 37.6 | 30.8-98.9 | 0.888 | 0.753-1.046 | 0.155 | -0.119 | 0.084 | 5.8 | 0.673 |  |  |
|  | Weighted median | 9 | 37.6 | 30.8-98.9 | 0.592 | 0.319-1.101 | 0.142 | -0.524 | 0.316 | 4.0 | 0.779 |  |  |
|  | MR Egger | 9 | 37.6 | 30.8-98.9 | 0.898 | 0.718-1.123 | 0.345 | -0.108 | 0.114 | NA | NA | 0.105 | 0.226 |
|  | Weighted mode | 9 | 37.6 | 30.8-98.9 | 0.807 | 0.555-1.173 | 0.293 | -0.215 | 0.191 | NA | NA |  |  |
|  | MR PRESSO | NA |  |  |  |  |  |  |  |  |  |  |  |
| IEAA age acceleration | Inverse variance weighted | 24 | 47.4 | 31.1-239.7 | 1.009 | 0.929-1.096 | 0.829 | 0.009 | 0.042 | 21.2 | 0.569 |  |  |
|  | Weighted median | 24 | 47.4 | 31.1-239.7 | 0.923 | 0.819-1.040 | 0.188 | -0.080 | 0.061 | NA | NA |  |  |
|  | MR Egger | 24 | 47.4 | 31.1-239.7 | 0.861 | 0.710-1.045 | 0.144 | -0.149 | 0.099 | 18.0 | 0.704 | 0.050 | 0.089 |
|  | Weighted mode | 24 | 47.4 | 31.1-239.7 | 0.923 | 0.794-1.074 | 0.312 | -0.080 | 0.077 | NA | NA |  |  |
|  | MR PRESSO | NA |  |  |  |  |  |  |  |  |  |  |  |
| PhenoAge acceleration | Inverse variance weighted | 11 | 44.9 | 31.8-89.4 | 0.963 | 0.864-1.073 | 0.494 | -0.038 | 0.055 | 10.4 | 0.404 |  |  |
|  | MR Egger | 11 | 44.9 | 31.8-89.4 | 0.750 | 0.542-1.037 | 0.116 | -0.288 | 0.166 | 7.9 | 0.547 |  |  |
|  | Weighted median | 11 | 44.9 | 31.8-89.4 | 0.911 | 0.795-1.045 | 0.183 | -0.093 | 0.070 | NA | NA | 0.095 | 0.144 |
|  | Weighted mode | 11 | 44.9 | 31.8-89.4 | 0.895 | 0.730-1.097 | 0.310 | -0.111 | 0.104 | NA | NA |  |  |
|  | MR PRESSO | NA |  |  |  |  |  |  |  |  |  |  |  |
| GrimAge acceleration | Inverse variance weighted | 4 | 35.9 | 31.1-45.5 | 1.173 | 0.782-1.760 | 0.441 | 0.160 | 0.207 | 7.2 | 0.067 |  |  |
|  | Weighted median | 4 | 35.9 | 31.1-45.5 | 1.071 | 0.766-1.499 | 0.688 | 0.069 | 0.171 | NA | NA |  |  |
|  | MR Egger | 4 | 35.9 | 31.1-45.5 | 1.550 | 0.000-6722.466 | 0.928 | 0.438 | 4.273 | 7.2 | **0.028** | -0.052 | 0.954 |
|  | Weighted mode | 4 | 35.9 | 31.1-45.5 | 0.927 | 0.503-1.709 | 0.825 | -0.075 | 0.312 | NA | NA |  |  |
|  | MR PRESSO | NA |  |  |  |  |  |  |  |  |  |  |  |
| Decrease in mtDNA copy number | Inverse variance weighted | 88 | 46.7 | 29.8-634.2 | 1.182 | 0.687-2.031 | 0.546 | 0.167 | 0.276 | 96.9 | 0.219 |  |  |
|  | Weighted median | 88 | 46.7 | 29.8-634.2 | 1.590 | 0.697-3.628 | 0.271 | 0.464 | 0.421 | NA | NA |  |  |
|  | MR Egger | 88 | 46.7 | 29.8-634.2 | 1.463 | 0.519-4.128 | 0.474 | 0.381 | 0.529 | 96.7 | 0.202 | -0.006 | 0.636 |
|  | Weighted mode | 88 | 46.7 | 29.8-634.2 | 1.534 | 0.562-4.185 | 0.406 | 0.428 | 0.512 | NA | NA |  |  |
|  | MR PRESSO | NA |  |  |  |  |  |  |  |  |  |  |  |
| BioAgeAcceleration (clinical) | Inverse variance weighted | 18 | 42.7 | 33.0-114.2 | 1.285 | 0.799-2.066 | 0.301 | 0.251 | 0.242 | 25.1 | 0.092 |  |  |
|  | Weighted median | 18 | 42.7 | 33.0-114.2 | 1.156 | 0.631-2.119 | 0.639 | 0.145 | 0.309 | NA | NA |  |  |
|  | MR Egger | 18 | 42.7 | 33.0-114.2 | 0.502 | 0.156-1.614 | 0.265 | -0.688 | 0.596 | 21.2 | 0.170 | 0.081 | 0.107 |
|  | Weighted mode | 18 | 42.7 | 33.0-114.2 | 0.958 | 0.357-2.569 | 0.932 | -0.043 | 0.504 | NA | NA |  |  |
|  | MR PRESSO | NA |  |  |  |  |  |  |  |  |  |  |  |
| PhenoAgeclAcceleration (clinical) | Inverse variance weighted | 55 | 46.5 | 30.4-371.7 | 0.892 | 0.756-1.053 | 0.177 | -0.114 | 0.084 | 214.2 | **0.000** |  |  |
|  | Weighted median | 55 | 46.5 | 30.4-371.7 | 0.954 | 0.836-1.088 | 0.479 | -0.048 | 0.067 | NA | NA |  |  |
|  | MR Egger | 55 | 46.5 | 30.4-371.7 | 0.329 | 0.213-0.509 | **0.000** | -1.111 | 0.222 | 150.3 | **0.000** | 0.190 | **0.000** |
|  | Weighted mode | 55 | 46.5 | 30.4-371.7 | 0.912 | 0.703-1.183 | 0.489 | -0.093 | 0.133 | NA | NA |  |  |
|  | MR PRESSO | NA |  |  |  |  |  |  |  |  |  |  |  |
| Decrease in Handgrip (right) | Inverse variance weighted | 163 | 38.1 | 29.7-231.7 | 1.883 | 0.822-4.312 | 0.134 | 0.633 | 0.423 | 193.0 | **0.048** |  |  |
|  | Weighted median | 163 | 38.1 | 29.7-231.7 | 1.054 | 0.327-3.394 | 0.930 | 0.052 | 0.597 | NA | NA |  |  |
|  | MR Egger | 163 | 38.1 | 29.7-231.7 | 3.096 | 0.140-68.526 | 0.476 | 1.130 | 1.580 | 192.9 | **0.044** | -0.006 | 0.744 |
|  | Weighted mode | 163 | 38.1 | 29.7-231.7 | 0.240 | 0.020-2.943 | 0.266 | -1.428 | 1.279 | NA | NA |  |  |
|  | MR PRESSO | NA |  |  |  |  |  |  |  |  |  |  |  |
| Decrease in Handgrip (left) | Inverse variance weighted | 144 | 39.0 | 29.7-191.5 | 2.183 | 0.971-4.911 | 0.059 | 0.781 | 0.414 | 142.0 | 0.509 |  |  |
|  | Weighted median | 144 | 39.0 | 29.7-191.5 | 1.925 | 0.571-6.486 | 0.291 | 0.655 | 0.620 | NA | NA |  |  |
|  | MR Egger | 144 | 39.0 | 29.7-191.5 | 1.676 | 0.077-36.290 | 0.743 | 0.516 | 1.569 | 141.9 | 0.486 | 0.003 | 0.862 |
|  | Weighted mode | 144 | 39.0 | 29.7-191.5 | 0.193 | 0.011-3.251 | 0.255 | -1.646 | 1.441 | NA | NA |  |  |
|  | MR PRESSO | NA |  |  |  |  |  |  |  |  |  |  |  |
| Decrease in Appendicular lean mass | Inverse variance weighted | 622 | 63.7 | 29.1-1072.6 | 1.129 | 0.915-1.392 | 0.257 | 0.121 | 0.107 | 625.9 | 0.438 |  |  |
|  | Weighted median | 622 | 63.7 | 29.1-1072.6 | 1.007 | 0.722-1.404 | 0.968 | 0.007 | 0.170 | NA | NA |  |  |
|  | MR Egger | 622 | 63.7 | 29.1-1072.6 | 0.912 | 0.562-1.482 | 0.711 | -0.092 | 0.248 | 624.9 | 0.437 | 0.005 | 0.340 |
|  | Weighted mode | 622 | 63.7 | 29.1-1072.6 | 0.895 | 0.440-1.819 | 0.759 | -0.111 | 0.362 | NA | NA |  |  |
|  | MR PRESSO | NA |  |  |  |  |  |  |  |  |  |  |  |
| Decrease in Lung function (FVC) | Inverse variance weighted | 304 | 45.6 | 29.9-456.6 | 1.265 | 0.801-1.997 | 0.313 | 0.235 | 0.233 | 334.2 | 0.105 |  |  |
|  | Weighted median | 304 | 45.6 | 29.9-456.6 | 1.127 | 0.558-2.274 | 0.739 | 0.119 | 0.358 | NA | NA |  |  |
|  | MR Egger | 304 | 45.6 | 29.9-456.6 | 1.248 | 0.352-4.418 | 0.732 | 0.221 | 0.645 | 334.2 | 0.098 | 0.000 | 0.982 |
|  | Weighted mode | 304 | 45.6 | 29.9-456.6 | 0.607 | 0.103-3.582 | 0.582 | -0.499 | 0.906 | NA | NA |  |  |
|  | MR PRESSO | NA |  |  |  |  |  |  |  |  |  |  |  |
| Decrease in Lung function (FEV1) | Inverse variance weighted | 251 | 44.2 | 29.7-270.6 | 1.100 | 0.648-1.869 | 0.724 | 0.096 | 0.270 | 304.5 | **0.010** |  |  |
|  | Weighted median | 251 | 44.2 | 29.7-270.6 | 1.107 | 0.535-2.290 | 0.785 | 0.101 | 0.371 | NA | NA |  |  |
|  | MR Egger | 251 | 44.2 | 29.7-270.6 | 0.816 | 0.160-4.167 | 0.807 | -0.204 | 0.832 | 304.3 | **0.010** | 0.005 | 0.704 |
|  | Weighted mode | 251 | 44.2 | 29.7-270.6 | 0.366 | 0.045-2.950 | 0.346 | -1.004 | 1.064 | NA | NA |  |  |
|  | MR PRESSO | 250 |  |  | 1.097 | 0.663-1.815 | 0.719 | 0.093 | 0.257 |  |  |  |  |
| Retinal eye clock | Inverse variance weighted | 10 | 32.6 | 28.0-217.7 | 1.098 | 0.921-1.309 | 0.298 | 0.094 | 0.090 | 4.9 | 0.839 |  |  |
|  | Weighted median | 10 | 32.6 | 28.0-217.7 | 1.055 | 0.843-1.320 | 0.641 | 0.053 | 0.114 | NA | NA |  |  |
|  | MR Egger | 10 | 32.6 | 28.0-217.7 | 0.947 | 0.600-1.494 | 0.819 | -0.055 | 0.233 | 4.5 | 0.812 | 0.035 | 0.509 |
|  | Weighted mode | 10 | 32.6 | 28.0-217.7 | 1.047 | 0.818-1.339 | 0.726 | 0.046 | 0.126 | NA | NA |  |  |
|  | MR PRESSO | NA |  |  |  |  |  |  |  |  |  |  |  |
| Decrease in Cognitive performance | Inverse variance weighted | 130 | 37.7 | 29.8-116.1 | 1.042 | 0.655-1.657 | 0.862 | 0.041 | 0.237 | 126.6 | 0.543 |  |  |
|  | Weighted median | 130 | 37.7 | 29.8-116.1 | 1.547 | 0.780-3.069 | 0.212 | 0.436 | 0.349 | NA | NA |  |  |
|  | MR Egger | 130 | 37.7 | 29.8-116.1 | 1.029 | 0.106-10.003 | 0.980 | 0.029 | 1.160 | 126.6 | 0.518 | 0.000 | 0.991 |
|  | Weighted mode | 130 | 37.7 | 29.8-116.1 | 2.549 | 0.417-15.575 | 0.313 | 0.936 | 0.923 | NA | NA |  |  |
|  | MR PRESSO | NA |  |  |  |  |  |  |  |  |  |  |  |
| Brain Age Gap | Inverse variance weighted | 7 | 33.1 | 30.3-94.3 | 0.854 | 0.647-1.126 | 0.263 | -0.158 | 0.141 | 7.8 | 0.251 |  |  |
|  | Weighted median | 7 | 33.1 | 30.3-94.3 | 1.005 | 0.720-1.404 | 0.976 | 0.005 | 0.170 | NA | NA |  |  |
|  | MR Egger | 7 | 33.1 | 30.3-94.3 | 0.314 | 0.100-0.981 | 0.103 | -1.159 | 0.582 | 4.7 | 0.450 | 0.194 | 0.138 |
|  | Weighted mode | 7 | 33.1 | 30.3-94.3 | 1.072 | 0.682-1.684 | 0.774 | 0.069 | 0.231 | NA | NA |  |  |
|  | MR PRESSO | NA |  |  |  |  |  |  |  |  |  |  |  |
| Frailty | Inverse variance weighted | 14 | 32.9 | 30.0-119.1 | 1.322 | 0.342-5.116 | 0.686 | 0.279 | 0.691 | 15.3 | 0.288 |  |  |
|  | Weighted median | 14 | 32.9 | 30.0-119.1 | 2.861 | 0.481-17.026 | 0.248 | 1.051 | 0.910 | NA | NA |  |  |
|  | MR Egger | 14 | 32.9 | 30.0-119.1 | 3.192 | 0.005-1867.098 | 0.727 | 1.161 | 3.251 | 15.2 | 0.230 | -0.020 | 0.786 |
|  | Weighted mode | 14 | 32.9 | 30.0-119.1 | 5.166 | 0.469-56.931 | 0.203 | 1.642 | 1.224 | NA | NA |  |  |
|  | MR PRESSO | NA |  |  |  |  |  |  |  |  |  |  |  |

**The association between biomarkers of aging and ischemic stroke**

| Exposure | method | SNP | medianF | F range | OR | 95%CI | P val | Beta | se | Q | P_Q_ | Egger intercept | P_intercept_ |
| --- | --- | --- | --- | --- | --- | --- | --- | --- | --- | --- | --- | --- | --- |
| Decrease in Telomere length | Inverse variance weighted | 138 | 59.3 | 29.8-1105.8 | 1.029 | 0.931-1.139 | 0.572 | 0.029 | 0.051 | 210.9 | **0.000** |  |  |
|  | Weighted median | 138 | 59.3 | 29.8-1105.8 | 1.020 | 0.897-1.160 | 0.763 | 0.020 | 0.066 | NA | NA |  |  |
|  | MR Egger | 138 | 59.3 | 29.8-1105.8 | 1.069 | 0.884-1.292 | 0.491 | 0.067 | 0.097 | 210.5 | **0.000** | -0.001 | 0.646 |
|  | Weighted mode | 138 | 59.3 | 29.8-1105.8 | 1.000 | 0.846-1.183 | 0.996 | 0.000 | 0.085 | NA | NA |  |  |
|  | MR PRESSO | 137 |  |  | 1.003 | 0.919-1.093 | 0.954 | 0.003 | 0.044 |  |  |  |  |
| Hannum age acceleration | Inverse variance weighted | 8 | 36.2 | 30.8-98.9 | 0.998 | 0.950-1.048 | 0.932 | -0.002 | 0.025 | 15.2 | **0.033** |  |  |
|  | Weighted median | 8 | 36.2 | 30.8-98.9 | 0.998 | 0.954-1.045 | 0.939 | -0.002 | 0.023 | NA | NA |  |  |
|  | MR Egger | 8 | 36.2 | 30.8-98.9 | 0.830 | 0.734-0.938 | 0.024 | -0.187 | 0.062 | 5.8 | 0.448 | 0.049 | **0.022** |
|  | Weighted mode | 8 | 36.2 | 30.8-98.9 | 0.994 | 0.943-1.048 | 0.837 | -0.006 | 0.027 | NA | NA |  |  |
|  | MR PRESSO | NA |  |  |  |  |  |  |  |  |  |  |  |
| IEAA age acceleration | Inverse variance weighted | 24 | 47.4 | 31.1-239.7 | 1.005 | 0.981-1.030 | 0.680 | 0.005 | 0.013 | 43.7 | **0.006** |  |  |
|  | Weighted median | 24 | 47.4 | 31.1-239.7 | 1.017 | 0.990-1.045 | 0.214 | 0.017 | 0.014 | NA | NA |  |  |
|  | MR Egger | 24 | 47.4 | 31.1-239.7 | 1.041 | 0.975-1.112 | 0.242 | 0.041 | 0.034 | 41.3 | **0.008** | -0.010 | 0.271 |
|  | Weighted mode | 24 | 47.4 | 31.1-239.7 | 1.029 | 0.990-1.070 | 0.155 | 0.029 | 0.020 | NA | NA |  |  |
|  | MR PRESSO | 23 |  |  | 1.012 | 0.995-1.030 | 0.189 | 0.012 | 0.009 |  |  |  |  |
| PhenoAge acceleration | Inverse variance weighted | 11 | 44.9 | 31.8-89.4 | 1.002 | 0.979-1.025 | 0.871 | 0.002 | 0.012 | 10.9 | 0.366 |  |  |
|  | Weighted median | 11 | 44.9 | 31.8-89.4 | 0.995 | 0.966-1.026 | 0.755 | -0.005 | 0.015 | NA | NA |  |  |
|  | MR Egger | 11 | 44.9 | 31.8-89.4 | 1.041 | 0.975-1.111 | 0.260 | 0.040 | 0.033 | 9.3 | 0.406 | -0.015 | 0.254 |
|  | Weighted mode | 11 | 44.9 | 31.8-89.4 | 0.992 | 0.941-1.046 | 0.776 | -0.008 | 0.027 | NA | NA |  |  |
|  | MR PRESSO | NA |  |  |  |  |  |  |  |  |  |  |  |
| GrimAge acceleration | Inverse variance weighted | 3 | 33.1 | 31.1-45.5 | 0.986 | 0.921-1.056 | 0.689 | -0.014 | 0.035 | 2.4 | 0.305 |  |  |
|  | Weighted median | 3 | 33.1 | 31.1-45.5 | 0.985 | 0.913-1.064 | 0.709 | -0.015 | 0.039 | NA | NA |  |  |
|  | MR Egger | 3 | 33.1 | 31.1-45.5 | 0.584 | 0.200-1.702 | 0.505 | -0.538 | 0.546 | 1.2 | 0.266 | 0.096 | 0.512 |
|  | Weighted mode | 3 | 33.1 | 31.1-45.5 | 0.973 | 0.878-1.077 | 0.648 | -0.028 | 0.052 | NA | NA |  |  |
|  | MR PRESSO | NA |  |  |  |  |  |  |  |  |  |  |  |
| Decreae in mtDNA copy number | Inverse variance weighted | 88 | 45.9 | 29.8-634.2 | 0.930 | 0.809-1.069 | 0.307 | -0.073 | 0.071 | 144.1 | **0.000** |  |  |
|  | Weighted median | 88 | 45.9 | 29.8-634.2 | 0.877 | 0.731-1.052 | 0.156 | -0.132 | 0.093 | NA | NA |  |  |
|  | MR Egger | 88 | 45.9 | 29.8-634.2 | 0.713 | 0.541-0.939 | **0.018** | -0.339 | 0.141 | 136.6 | **0.000** | 0.007 | **0.032** |
|  | Weighted mode | 88 | 45.9 | 29.8-634.2 | 0.817 | 0.640-1.044 | 0.110 | -0.202 | 0.125 | NA | NA |  |  |
|  | MR PRESSO | 86 |  |  | 0.918 | 0.812-1.038 | 0.176 | -0.085 | 0.063 |  |  |  |  |
| BioAgeAccdeleration (clinical) | Inverse variance weighted | 19 | 42.7 | 33.0-270.1 | 1.296 | 1.194-1.406 | **0.000** | 0.259 | 0.042 | 22.7 | 0.201 |  |  |
|  | Weighted median | 19 | 42.7 | 33.0-270.1 | 1.281 | 1.148-1.428 | **0.000** | 0.247 | 0.056 | NA | NA |  |  |
|  | MR Egger | 19 | 42.7 | 33.0-270.1 | 1.175 | 0.990-1.395 | 0.082 | 0.161 | 0.087 | 20.8 | 0.237 | 0.009 | 0.222 |
|  | Weighted mode | 19 | 42.7 | 33.0-270.1 | 1.244 | 1.069-1.447 | **0.011** | 0.218 | 0.077 | NA | NA |  |  |
|  | MR PRESSO | NA |  |  |  |  |  |  |  |  |  |  |  |
| PhenoAgecAccedleration (clinical) | Inverse variance weighted | 55 | 46.3 | 30.4-371.7 | 1.023 | 1.001-1.046 | **0.039** | 0.023 | 0.011 | 87.9 | **0.002** |  |  |
|  | Weighted median | 55 | 46.3 | 30.4-371.7 | 1.017 | 0.990-1.046 | 0.214 | 0.017 | 0.014 | NA | NA |  |  |
|  | MR Egger | 55 | 46.3 | 30.4-371.7 | 1.037 | 0.971-1.108 | 0.283 | 0.036 | 0.034 | 87.6 | **0.002** | -0.003 | 0.675 |
|  | Weighted mode | 55 | 46.3 | 30.4-371.7 | 1.015 | 0.969-1.063 | 0.539 | 0.015 | 0.024 | NA | NA |  |  |
|  | MR PRESSO | 54 |  |  | 1.027 | 1.006-1.048 | **0.014** | 0.026 | 0.010 |  |  |  |  |
| Decrease in Handgrip (right) | Inverse variance weighted | 163 | 37.9 | 29.7-231.7 | 1.023 | 0.856-1.223 | 0.799 | 0.023 | 0.091 | 210.2 | **0.006** |  |  |
|  | Weighted median | 163 | 37.9 | 29.7-231.7 | 1.002 | 0.794-1.264 | 0.990 | 0.002 | 0.119 | NA | NA |  |  |
|  | MR Egger | 163 | 37.9 | 29.7-231.7 | 0.923 | 0.462-1.843 | 0.821 | -0.080 | 0.353 | 210.0 | **0.006** | 0.001 | 0.762 |
|  | Weighted mode | 163 | 37.9 | 29.7-231.7 | 1.102 | 0.588-2.066 | 0.763 | 0.097 | 0.321 | NA | NA |  |  |
|  | MR PRESSO | 162 |  |  | 1.049 | 0.888-1.240 | 0.573 | 0.048 | 0.085 |  |  |  |  |
| Decrease in Handgrip (left) | Inverse variance weighted | 147 | 38.9 | 29.7-191.5 | 1.081 | 0.917-1.273 | 0.353 | 0.078 | 0.083 | 145.5 | 0.497 |  |  |
|  | Weighted median | 147 | 38.9 | 29.7-191.5 | 1.130 | 0.878-1.453 | 0.343 | 0.122 | 0.128 | NA | NA |  |  |
|  | MR Egger | 147 | 38.9 | 29.7-191.5 | 0.979 | 0.519-1.848 | 0.949 | -0.021 | 0.324 | 145.4 | 0.476 | 0.001 | 0.754 |
|  | Weighted mode | 147 | 38.9 | 29.7-191.5 | 0.703 | 0.344-1.437 | 0.336 | -0.352 | 0.365 | NA | NA |  |  |
|  | MR PRESSO | NA |  |  |  |  |  |  |  |  |  |  |  |
| Decrease in Appendicular Lean mass | Inverse variance weighted | 639 | 64.0 | 29.1-1072.6 | 1.077 | 1.023-1.134 | **0.005** | 0.074 | 0.026 | 928.1 | **0.000** |  |  |
|  | Weighted median | 639 | 64.0 | 29.1-1072.6 | 1.038 | 0.966-1.115 | 0.311 | 0.037 | 0.037 | NA | NA |  |  |
|  | MR Egger | 639 | 64.0 | 29.1-1072.6 | 1.045 | 0.925-1.179 | 0.480 | 0.044 | 0.062 | 927.6 | **0.000** | 0.001 | 0.587 |
|  | Weighted mode | 639 | 64.0 | 29.1-1072.6 | 1.003 | 0.845-1.190 | 0.976 | 0.003 | 0.088 | NA | NA |  |  |
|  | MR PRESSO | 637 |  |  | 1.061 | 1.011-1.114 | **0.017** | 0.059 | 0.025 |  |  |  |  |
| Decrease in Lung function (FVC) | Inverse variance weighted | 309 | 45.5 | 29.9-456.6 | 1.120 | 1.006-1.248 | **0.039** | 0.113 | 0.055 | 463.9 | **0.000** |  |  |
|  | Weighted median | 309 | 45.5 | 29.9-456.6 | 1.137 | 0.989-1.308 | 0.072 | 0.129 | 0.071 | NA | NA |  |  |
|  | MR Egger | 309 | 45.5 | 29.9-456.6 | 1.139 | 0.843-1.541 | 0.397 | 0.130 | 0.154 | 463.8 | **0.000** | -0.000 | 0.906 |
|  | Weighted mode | 309 | 45.5 | 29.9-456.6 | 1.180 | 0.829-1.680 | 0.359 | 0.165 | 0.180 | NA | NA |  |  |
|  | MR PRESSO | 306 |  |  | 1.049 | 0.888-1.240 | **0.049** | 0.102 | 0.051 |  |  |  |  |
| Decrease in Lung function (FEV1) | Inverse variance weighted | 253 | 44.2 | 29.7-270.6 | 1.159 | 1.027-1.309 | **0.017** | 0.148 | 0.062 | 390.7 | **0.000** |  |  |
|  | Weighted median | 253 | 44.2 | 29.7-270.6 | 1.150 | 0.979-1.352 | 0.088 | 0.140 | 0.082 | NA | NA |  |  |
|  | MR Egger | 253 | 44.2 | 29.7-270.6 | 1.137 | 0.778-1.662 | 0.508 | 0.128 | 0.194 | 390.7 | **0.000** | 0.000 | 0.915 |
|  | Weighted mode | 253 | 44.2 | 29.7-270.6 | 1.206 | 0.807-1.802 | 0.362 | 0.187 | 0.205 | NA | NA |  |  |
|  | MR PRESSO | 250 |  |  | 1.166 | 1.042-1.306 | **0.008** | 0.154 | 0.058 |  |  |  |  |
| Retinal eye clock | Inverse variance weighted | 11 | 32.6 | 28.0-217.7 | 1.025 | 0.970-1.084 | 0.379 | 0.025 | 0.028 | 26.8 | **0.003** |  |  |
|  | Weighted median | 11 | 32.6 | 28.0-217.7 | 0.962 | 0.829-1.116 | 0.619 | -0.039 | 0.076 | 24.5 | **0.004** |  |  |
|  | MR Egger | 11 | 32.6 | 28.0-217.7 | 1.003 | 0.958-1.051 | 0.884 | 0.003 | 0.024 | NA | NA | 0.015 | 0.387 |
|  | Weighted mode | 11 | 32.6 | 28.0-217.7 | 1.009 | 0.960-1.061 | 0.737 | 0.009 | 0.026 | NA | NA |  |  |
|  | MR PRESSO |  |  |  |  |  |  |  |  |  |  |  |  |
| Decrease in Cognitive performance | Inverse variance weighted | 132 | 38.0 | 29.8-125.3 | 1.061 | 0.963-1.169 | 0.229 | 0.059 | 0.049 | 142.9 | 0.225 |  |  |
|  | Weighted median | 132 | 38.0 | 29.8-125.3 | 1.059 | 0.925-1.213 | 0.405 | 0.058 | 0.069 | NA | NA |  |  |
|  | MR Egger | 132 | 38.0 | 29.8-125.3 | 1.102 | 0.711-1.708 | 0.666 | 0.097 | 0.224 | 142.9 | 0.208 | -0.001 | 0.864 |
|  | Weighted mode | 132 | 38.0 | 29.8-125.3 | 1.099 | 0.792-1.525 | 0.574 | 0.094 | 0.167 | NA | NA |  |  |
|  | MR PRESSO | NA |  |  |  |  |  |  |  |  |  |  |  |
| Brain Age Gap | Inverse variance weighted | 7 | 33.1 | 30.3-94.3 | 0.986 | 0.912-1.065 | 0.717 | -0.014 | 0.039 | 17.1 | **0.009** |  |  |
|  | Weighted median | 7 | 33.1 | 30.3-94.3 | 1.007 | 0.944-1.076 | 0.826 | 0.007 | 0.033 | NA | NA |  |  |
|  | MR Egger | 7 | 33.1 | 30.3-94.3 | 1.036 | 0.721-1.491 | 0.855 | 0.036 | 0.185 | 16.8 | **0.005** | -0.010 | 0.793 |
|  | Weighted mode | 7 | 33.1 | 30.3-94.3 | 1.024 | 0.935-1.121 | 0.628 | 0.024 | 0.046 | NA | NA |  |  |
|  | MR PRESSO |  |  |  |  |  |  |  |  |  |  |  |  |
| Frailty | Inverse variance weighted | 13 | 32.6 | 30.0-40.6 | 1.420 | 1.069-1.887 | **0.016** | 0.351 | 0.145 | 7.5 | 0.826 |  |  |
|  | Weighted median | 13 | 32.6 | 30.0-40.6 | 1.345 | 0.929-1.946 | 0.116 | 0.296 | 0.189 | NA | NA |  |  |
|  | MR Egger | 13 | 32.6 | 30.0-40.6 | 1.263 | 0.066-24.306 | 0.880 | 0.234 | 1.509 | 7.4 | 0.762 | 0.002 | 0.939 |
|  | Weighted mode | 13 | 32.6 | 30.0-40.6 | 1.293 | 0.704-2.376 | 0.423 | 0.257 | 0.310 | NA | NA |  |  |
|  | MR PRESSO | NA |  |  |  |  |  |  |  |  |  |  |  |

**Supplementary table 3: Power calculation MR**

IVW-MR estimates were calculated using an online MR power calculation tool

<https://sb452.shinyapps.io/power/>

AD: Sample size required for 80% power

|  | Actual Sample size  (# of participants) | Power (%) | Case: control  1:x | R^2^ | Causal effect per SD change | Significant level | **Sample size required for 80 % power** |
| --- | --- | --- | --- | --- | --- | --- | --- |
| TL | 85934+401577  =487,511 | 80% | 1:4.7 | 0.0333 | 1.1 | 0.05 | 181,000 |
| Hannum |  |  |  | 0.0148 |  |  | 403,600 |
| IEAA |  |  |  | 0.0441 |  |  | 135,500 |
| PhenoAge |  |  |  | 0.0186 |  |  | 321,200 |
| GrimAge |  |  |  | 0.0047 |  |  | **1270,900** |
| mtDNA CN |  |  |  | 0.0182 |  |  | 328,200 |
| BioAge Acc |  |  |  | 0.0093 |  |  | **642,300** |
| PhenoAge Acc |  |  |  | 0.0261 |  |  | 228,900 |
| Handgrip R |  |  |  | 0.0168 |  |  | 355,600 |
| Handgrip L |  |  |  | 0.0151 |  |  | 395,600 |
| Lean mass |  |  |  | 0.1350 |  |  | 44300 |
| FVC |  |  |  | 0.0458 |  |  | 130,500 |
| FEV1 |  |  |  | 0.0335 |  |  | 178,300 |
| Retinal age |  |  |  | 0.0122 |  |  | **489,600** |
| Cognitive performance |  |  |  | 0.0224 |  |  | 266,700 |
| BAG |  |  |  | 0.0117 |  |  | **510,500** |
| Frailty index |  |  |  | 0.0032 |  |  | **1866,600** |

VaD: Sample size required for 80% power

|  | Actual Sample size | Power (%) | Case: control  1:x | R^2 | Causal effect per SD change | Significant level | **Sample size required for 80% power** |
| --- | --- | --- | --- | --- | --- | --- | --- |
| TL | 2048+328,982  =331,030 | 80% | 160.64 | 0.0333 | 1.1 | 0.05 | **4219,200** |
| Hannum |  |  |  | 0.0148 |  |  | **9493,100** |
| IEAA |  |  |  | 0.0441 |  |  | **3187,700** |
| PhenoAge |  |  |  | 0.0186 |  |  | **7555,500** |
| GrimAge |  |  |  | 0.0047 |  |  | **29900,300** |
| mtDNA CN |  |  |  | 0.0182 |  |  | **7721,600** |
| BioAge Acc |  |  |  | 0.0093 |  |  | **15110,900** |
| PhenoAge Acc |  |  |  | 0.0261 |  |  | **5384,400** |
| Handgrip R |  |  |  | 0.0168 |  |  | **8365,000** |
| Handgrip L |  |  |  | 0.0151 |  |  | **9306,800** |
| Lean mass |  |  |  | 0.1350 |  |  | **1041,000** |
| FVC |  |  |  | 0.0458 |  |  | **3068,400** |
| FEV1 |  |  |  | 0.0335 |  |  | **4195,000** |
| Retinal age |  |  |  | 0.0122 |  |  | **11519,000** |
| Cognitive performance |  |  |  | 0.0224 |  |  | **6273,800** |
| BAG |  |  |  | 0.0117 |  |  | **12011,300** |
| Frailty index |  |  |  | 0.0032 |  |  | **43916,100** |
